# Exploring the feasibility of using school food purchase data as a method to assess dietary intakes in secondary school aged pupils

**DOI:** 10.1017/S1368980025000527

**Published:** 2025-04-14

**Authors:** Jennifer Bradley, Suzanne Spence

**Affiliations:** Human Nutrition and Exercise Research Centre, Population Health Sciences Institute, Newcastle University, Newcastle upon Tyne, UK

**Keywords:** Food purchase data, Dietary assessment, Secondary schools

## Abstract

**Objective:**

To explore the information available in school food purchase data and ascertain the potential to assess pupils’ dietary intakes. The proportion of purchased food and drink items that were linked to (i) an Intake24 food group and (ii) a nutrient code from the UK National Diet and Nutrition Survey (NDNS) Databank was calculated.

**Design:**

Pupil-level food purchase data covering the whole school day were obtained. Each item purchased was linked to an Intake24 food group and an NDNS Nutrient Databank code. Depending on the level of detail provided, items may have been assigned both a food group and a nutrient code, a food group only or neither for items, which did not contain enough information about the type of food or drink purchased.

**Setting:**

Five secondary schools in northeast England.

**Participants:**

Secondary school pupils aged 11–16 years.

**Results:**

The data captured 119 125 purchases made by 3466 pupils. 92 % of item descriptions were assigned a food group, and this equated to 82 % of total purchases. 70 % were assigned an NDNS Databank nutrient code, which accounted for 60 % of total purchases. 8 % of items had insufficient information and did not have a food group or a nutrient code assigned.

**Conclusions:**

The methodological challenges of collecting dietary data from pupils in the secondary school setting are significant. Purchase data offers an alternative, objective approach to collecting information on school food choices across the school day and for a large sample of pupils. With further development, the potential to use purchase data to assess intakes could be achieved.

A key public health priority is to improve children’s dietary intakes across the socio-economic spectrum. According to the UK National Diet and Nutrition Survey (NDNS), children are consuming too much free sugars, saturated fat and not enough fibre or fruits and vegetables^(1)^. School food contributes to 30 % of a child’s dietary intake, and therefore, school is an important setting for improving diet^(2)^. In the United Kingdom, approximately 57 % of secondary pupils consume a school meal at least 4 days per week. This compares to 16 % having a home-packed lunch and 28 % having a mixture of the two. Therefore, 85 % of pupils are purchasing a school lunch on at least one school day per week^(3)^.

In the United Kingdom, food-based standards apply to school lunch provision and foods available across the whole school day, to encourage a healthy balance of nutritional intake and restrict the availability of foods high in fat, salt and sugar^(4)^. However, there is currently no monitoring to check adherence to standards. A recent study found on average 64 % of standards were complied with in a sample of thirty-six secondary schools. No schools achieved 100 % compliance^(5)^. Urgent action is needed to improve school food for secondary pupils’, as evidence shows intakes of fruits and vegetables and protein-rich foods decrease from primary to secondary, and the proportion of sweet and savoury snacks increases^(6)^. It is vital that appropriate methods are available to assess the impact of policies, such as those to improve diet, in large-scale evaluations.

Self-reported methods, such as dietary recalls and food diaries, are often used to obtain information on pupils’ food and drink intakes in school. These methods are feasible to use in the school setting; however, they are costly to run and methodological challenges exist^(7,8)^. The subjective nature of these methods can lead to reporting errors due to memory, social desirability and motivation to complete; these issues are well documented and are a particular concern in the adolescent age group^(9–11)^. Advances in technology have led to the development of online tools that are more engaging for adolescents and have streamlined the process of dietary data collection^(10,12)^. However, reporting errors continue to be a challenge. In addition to methodological issues, gaining access into schools to conduct research can be difficult. Existing pressures on school staff mean research opportunities, although considered a benefit to schools, are often declined^(13)^.

An alternative approach to obtaining school food intake data may be the use of school food purchase data (SFPD). Individual-level SFPD provides information on the date and time of purchase, details of the food/drink item purchased, item cost and free school meal (FSM) status of the pupil purchasing the item. Utilising SFPD to explore pupils’ dietary intakes and patterns at school provides an alternative approach, where traditional methods are unfeasible, and simplifies the data collection procedure for both the researcher and the school. SFPD can be obtained without disruption to teaching and without the need for researchers to be present in school; therefore, reducing the burden on schools and pupils. Using SFPD to assess pupils’ dietary intakes will allow larger sample sizes of schools and pupils, covering the whole school day (including break), and across all levels of deprivation, whilst maintaining consistency of methods, fundamental for effective evaluation of policies.

Over the last decade, there has been a rapid increase in the use of commercial food purchase data, such as supermarket sales data, in public health nutrition research^(14)^. These data enable researchers to explore the dietary patterns of a large population. The information is objective, can have wide geographical coverage and is a useful indicator of dietary choices^(14)^. Food purchase data have been used to evaluate the impact of dietary interventions and national policies, such as sugar taxes and food assistance programs^(15,16)^. A recent systematic review concluded that supermarket sales data was useful for longitudinal dietary surveillance and can be used to better understand food purchase behaviours in high- and middle-income populations^(15)^. Jenneson *et al.* (2021) compared dietary intake estimates from supermarket transaction data with an FFQ and found the strongest agreement for single-person households and loyal customers^(17)^. It could therefore be hypothesised that dietary estimates derived from SFPD may provide good agreement with self-reported dietary intakes as items are usually purchased and consumed by the same individual. However, the level of detail available in SFPD and the ability to link to nutrient data warrants exploration. There is little published research exploring the use of SFPD as a method of estimating dietary intakes. The current study explored the procedures to obtain these data and the potential opportunities and limitations of using these data to estimate food and nutrient intakes in a sample of secondary schools in northeast England.

## Aims and objectives

The key aims were (i) to explore the use of secondary school pupils’ food purchase data as a potential method to estimate pupils’ food and drink intakes across the school day and (ii) explore the potential to link purchased items to food groups and food composition data. The objectives were:

- To ascertain the detail and variation in item descriptions (i.e. the description of the item purchased, e.g. ‘jacket potato’ or ‘toast’) contained in the data from the different schools and create overall item categories
- To determine the proportion of purchased food and drink items that could be linked to an Intake24 food group
- To determine the proportion of purchased food and drink items that could be linked to a food code from the UK NDNS Nutrient Databank (NDB)

## Methods

### Study setting and data collection

Existing links with a school trust facilitated purchase data capture (*n* 7 schools). Information about the study was shared with the school heads and the directors of IT and communications for the schools, and an opportunity to ask questions was given. It was emphasised that as SFPD are anonymised, no pupils would be identifiable. A 4-week data collection period (7^th^ November 2022 to 2^nd^ December 2022) was selected to take account of the 4-week school menu cycle and avoid school holiday periods and bank holidays. A token of thanks was given to the schools for their time.

In order to support the ethics application, a data management plan and a data protection impact assessment were created. The data protection impact assessment highlighted the requirement of a data sharing agreement (to be provided by the schools) and a privacy notice provided by the research team; the latter was necessary as the data was being used in a way that was not its original intended purpose.

An MS Excel spreadsheet (encrypted with a password) containing all school food purchase transactions across the whole school day for the 4-week period was sent to the research team via the school’s IT manager. The data included pupil purchase card number, date of purchase, time of purchase, item description (i.e. the food or drink purchased), quantity purchased, cost (£) of the item and whether the pupil received free school meals or not.

Due to access issues within the schools at the time of data download, data could not be captured for two of the schools; this was an internal IT issue and beyond the researchers’ control. However, the five schools included in the data represented a range of geographical locations.

### Data formatting

Data were formatted using MS Excel and Stata v18. Data from each school were merged into one dataset. Staff purchases and purchases made by pupils in years 12 and 13 were removed from the data as they are able to buy food and drink off-premises. Meal type (breakfast, break or lunch) was assigned to each food and drink item in the dataset according to the time it was purchased. Before 9 am was assigned ‘breakfast’, between 9 and 12 pm was assigned ‘break’ and 12 pm onwards was assigned ‘lunch’. These times were in accordance with the school timetable issued on the school websites.

Food and drink item descriptions in the dataset were derived from the labels on the till buttons operated by school canteen staff at the point of purchase. Due to variations in item descriptions across the schools, items were grouped into categories to combine similar items (see online supplementary material, Supplementary Material 1). These were created by the first researcher and agreed/reviewed by the second researcher.

### Linking purchased items with food composition data and food groups

**Figure 1.**
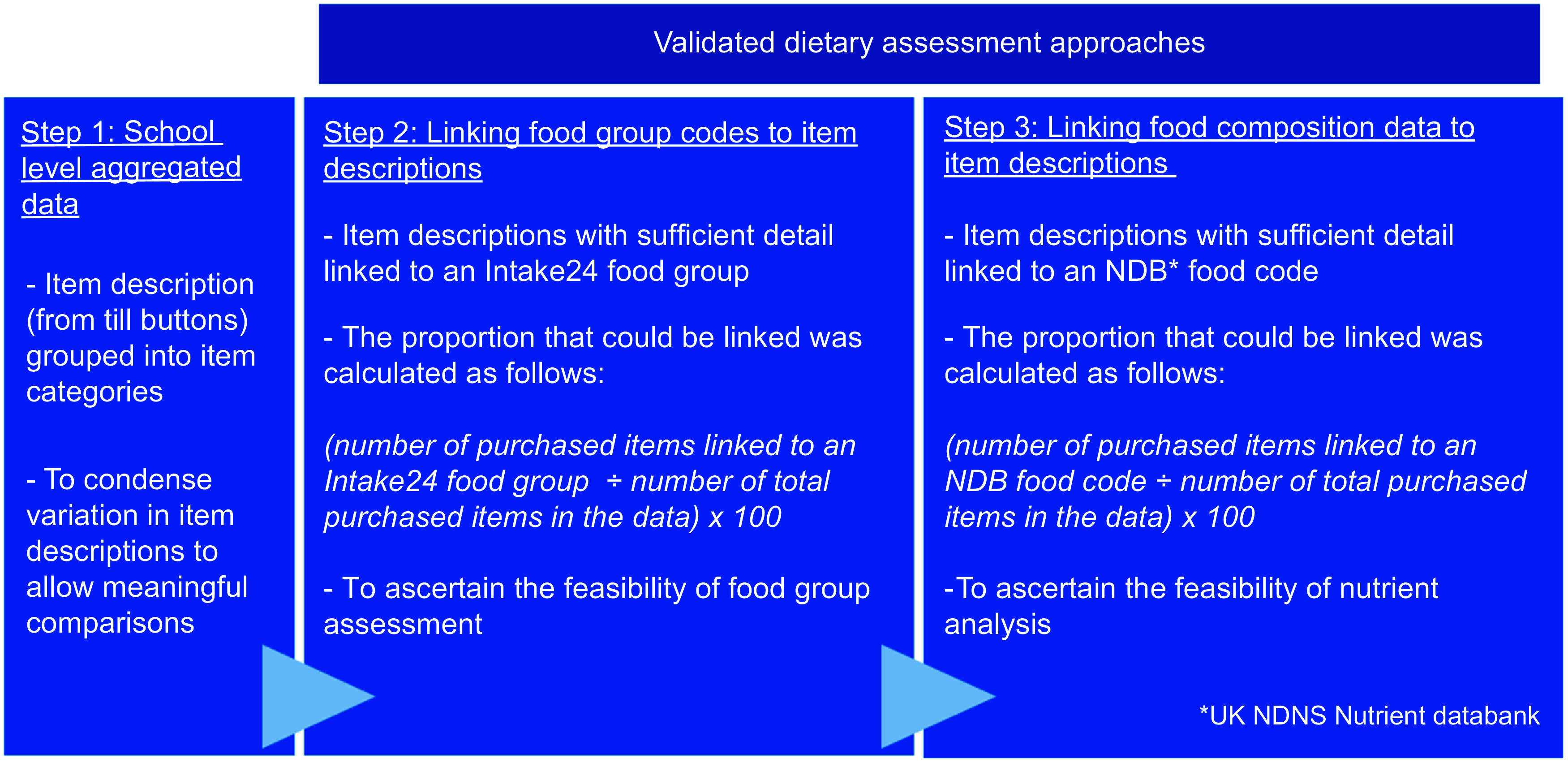
The process of data formatting and coding to ascertain the potential level of dietary assessment.

To explore the potential to link purchased items to food groups and food composition data and consider the level of analysis that could be performed, food and drink item descriptions in the data were coded at two levels (Fig. 1). Firstly, purchased food and drink items were linked to an Intake24 food group. These food groups are contained within the Intake24 system which is the dietary assessment tool used in the UK NDNS^(18)^. The proportion that could be assigned to a food group was calculated (see Fig. 1).

Secondly, purchased food and drink items were linked to a nutrient code from the UK NDNS NDB; the food composition database was developed for the UK NDNS^(18)^. Nutrient analysis could not be conducted as there was no portion size consumption data available. However, food and drink item descriptions in the data were matched with NDB nutrient codes to ascertain the potential of nutrient analysis using purchase data. For some main dishes, menus available from the school websites were referred to for clarification on the type of meal. For example, ‘Chinese noodles’ was a vegetarian option on the menu and was therefore coded as ‘*vegetable chow mein*’ (the closest match in the NDB), rather than a code with meat. The item description ‘Italian chicken’ was a pasta dish of the day and was therefore coded as ‘*pasta with meat in a tomato-based sauce’*. School menus were also checked for information about how the food was served. For example ‘meatball melt’ was served in a sub roll, which resulted in two NDB codes being assigned: ‘*meatballs in a tomato sauce*’ and ‘*rolls, white soft, not fortified*’. The proportion of items that could be linked to an NDB nutrient code was calculated. To assess inter-coder reliability, a second coder independently assigned NDB codes and food groups to a subset of item descriptions (20 %). The most commonly purchased items were selected, removing duplicates. The percentage agreement was calculated.

## Results

Pupil-level purchase data was obtained for five of the seven secondary schools. Refunded items (indicated by a negative cost value) were removed from the data; these only accounted for 0·3 % of total purchases. The data captured a total of 3466 pupils over a 4-week period in November 2022. Using figures available from local council websites, a total of 4330 pupils were enrolled across the five schools. Therefore, approximately 80 % of pupils purchased at least one food or drink item at school within the 4-week period.

### Variations in item descriptions

A total of 119 125 purchases were made by pupils at the five schools. In total, there were 367 different food and drink item descriptions in the dataset. These were derived from the labels on the till buttons in the canteen and varied by school. Identical items had slightly different item descriptions, which could be grouped together into item categories to allow meaningful comparisons. For example ‘*Fresh Fruit Pot*’, ‘*Fresh Fruit Salad*’, ‘*Fruit pot all*’ and ‘*Fruit Salad*’ were grouped into the item category ‘Fruit pot’. Similarly, ‘*Bacon & Sausage Bun*’, ‘*Bacon Bun*’, ‘*Bacon Sandwich Large*’, ‘*Bacon Small*’, ‘*Breakfast Bun*’ and ‘*Sausage bun*’ were all grouped into the category ‘Bacon/sausage bun’. The variation in how food and drink items were described across the five schools and all the categories created can be seen in online supplementary material, Supplementary Material 1.

### Linking with Intake24 food groups and Nutrient Databank food composition data

**Figure 2.**
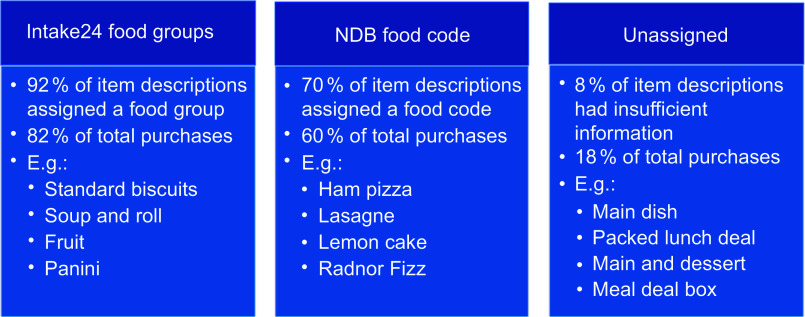
Proportion of food and drink item descriptions that were linked with Intake24 food groups, NDB food composition data and unassigned to either. NDB, Nutrient Databank.

Ninety-two percent (*n* 336) of item descriptions were assigned an Intake24 food group (Fig. 2). These accounted for 82 % (*n* 97 821) of total purchases. Of these, *n* 258 item descriptions contained enough information for an NDB nutrient code to be assigned (Table 1). These accounted for 60 % of total purchases. Some items (*n* 32) were linked to two or more food codes, for example, ‘meatballs & spag’ required both ‘*Meatballs in tomato sauce*’ and ‘*Pasta spaghetti boiled white*’. Similarly, the item description ‘beef stir fry & rice’ required two codes; ‘*Beef stir fry*’ and ‘*White rice basmati boiled*’. The coding scheme is provided in see online supplementary material, Supplementary Material 2.

**Table 1.** Item descriptions that were assigned both an Intake24 food group and a nutrient databank code, or a food group only, or neither

	Item descriptions	Proportion of total purchases (*n* (%))	
		*n*	%
Sufficient detail to assign an Intake24 food group^*^ and an NDB nutrient code^†^	Apple Cake; Apple Crumb; Apple Crumbl; Apple Crumble; Apple; Gammon Pineapple; Gammon steak; Bacon & Sausage Bun; Bacon Bun; Bacon Sandwich Large; Bacon Small; Bagel; Bagel HALF; Banana Cake; Banana Sponge; Banana; BBQ Sauce; BBQ Chicken Wrap; Cajun Chick Quasadil; Baked Beans; Beans on Toast; Mince & dumpling; Mince & Dumplings; Mince and Veg; Mince Beef Casserole; Beef Stir Fry & Rice; Roast Beef&York/Pud; Burger; Bolognaise; Bolognaise & G/Bread; Spicy potato; Bread bun; Crusty Roll; Poppy Seed Large Rol; Toast & Butter; Toast; Toast Main Hall; Extra Butter; Coke; Dr Pepper; Fanta; Suso Cans; Susu Can; Carrot Cake; Cauliflower Cheese; Cheese &Onion Pie; Cheese&Onion Quiche; Cheese & Tom Quiche; Cheese & Tom Pizza; Cheese Pizza; Cold Pizza Slice; Morning Pizza; Pizza - Hidden Sauce; Pizza bread; Pizza Large; Pizza Muffin; Pizza Small; Pizzini; Cheese; Toastie; Cheese Quesadilla; Cheese Scone; Cheese Cakes; Strawberry Cheesecak; Plain Chicken; Chick/Broc Pasta; Chicken Casserole; Harvest Chicken Cass; S/F Chicken; Cajun Chicken; Jerk chicken; Grill Chicken Burger; Tikka Masala & Rice; Chicken Korma; Chicken Tikka; Chicken Tikka Masala; Chicken Curry; Chicken Jalfrazi; Chicken Jalrezi; Sweet Chilli Enchil; BBQ Chicken pizza; BBQ Chicken; Sweet Chilli Chicken; Veg Chickpea Pocket; Veg Pea Pota Curry; Chilli Taco; Chilli-Con-Carni; Chilli beef taco; Chilli Beef Tacos; Portion of Chips; Muffin; Chocolate Bars; Cornflake Tart; Mousse; Chocolate orange; Choc & Orange cake; Chocolate Orang Cake; chocolate sponge; Pepporoni Panini; Cod and Chips; Fish & Chips; Coffee; Choc bis; Chocolate Crunch; Cottage Pie; Croissants; Crossiants; Crumpet; Crumpets; Dried Fruit Pot; Chicken fajita; Chicken Fajita Pocke; Chicken Fajitas; Salad bar plate; Salad Bowl; Salad Portion; Flapjack; Apple Flapjack; Berry Flapjack; Flavoured milk; Flavoured Milk 200 ml; Milkshake; Butter/Flora Ptn; Fresh Fruit Pot; Fresh Fruit Salad; Fruit pot all; Fruit Salad; Cherry Crumble; Radnor fizz; Radnor Fizz 300 ml; Oasis; Cuplet; Garlic Bread; Ginger Cake; Grapes; Tub Grapes; Ham; Hot Chocolate; Jam portion; Jelly; Jelly Squeeze; Jellys; Lamb Burger; Lasagne; Lasagne&Garlic Bread; Iced Cake; Lemon Cake; Lemon Muffin; Mayo Sauce; Meatball Melt; Meatballs & Spag; Melon; Melon fruit pot; Melon Pot; Milk; Milk carton; Mince Pie; Flavoured Water; Flavoured water 330 m; Rad Water 330 ml; Radnor Splash 500 ml; Juice Burst; Juice Burst 330 ml; Juiceburst; Juiceburst 330; Rad Frt Jc Car 125 ml; Tropicana juice; Carton Juice 220 ml; Fresh fruit carton; Fruit Carton; Fruit Juice Carton; Fruit Juice Cup; Tandoori Flat Bread; Tandoori flatbread; Orange; Chinese Chick Noodle; Chicken noodles; Pancakes; Plain Pasta & Cheese; Plain Pasta; Noodle Box; Chicken Italian; Italien Chicken; Pasta pot; Pasta Pot Tomato; Pasta with Sauce; Tomato Pasta; Pasta Arrabiata; Eton Mess; Pineapple fruit pot; Ham & Pin Pizza; Ham pizza; Proper Popcorn; Roast Pork Dinner; Roast Loin Pork; Meatballs; Spicy Meat Balls; Pork Sausage & Gravy; Sausage &Onion Gravy; Sausage & onion; Cumberland sausage; Sausage bun; Porridge; Crisps; propercrisps; Pringles; Half Jacket; Jacket potato/butter; Bacon/Cheese Jacket; Jacket Potato; Flora portion; Quorn Lasagne; Rice Pudding; Roast Beef Dinner; Chicken Dinner; Roast Chicken Dinner; Roast Turkey Dinner; Pepperoni; Sausage rolls; chilli pasta; Spaghi Bolognese; chicken wrap; Raspberry Coco Spong; Sponge Cake; Veg Spring Rolls; Sticky Date Pud; vegetable wrap; BBQ Veg Wrap; Veg Fajita; Veg Fajita Wrap; Vegetable Fajita Wra; Stuffed Peppers; Sweet n sour noodles; Sweet/Sour & Noodles; Sweet Chilli Sauces; Sweetcorn; Swiss Roll; Teacake; Toasted Tea Cake; Chicken Pasta Bake; Chicken tomat pasta; Chicken tomato pasta; Ketchup; Tuna; Pasta Pot Tuna; Roast Turkey; Chinese noodles; Veg Chinese Noodles; Quorn Korma; Mushroom Korma; Veg Bolognaise; Veg Burger; Plain Water 330 ml; Plain Water 500 ml; Water; water 330 ml; Frozen Yoghurt; Yoghurt	71 519	60 %
Sufficient detail to assign a food group only	Cereal; Fruit; Assorted Baguettes; Assorted Biscuits; Baguette; Baguette Meal Deal; Baked Potato Deal; Biscuit; Breakfast Bun; Cake & Custard; Cereal Bars; Cheese Savoury; Cheese tart; Cheese/Tuna; Chicken & Bacon; Classic Triangle S/W; Cold Baguettes; Extra Sauce; Extra veg portion; Filled Jacket 1 Fill; Fresh Fruit; Fruit; Fruit Flakes; Homebakes; Homemade Biscuits; Homemade Soup; Hot Meat Baguette; Jacket & cold Fill; Jacket Meal Deal; Jacket Pot & Filling; Jacket Pot Meal Deal; Jacket Pot&2 Filling; Jacket potato 1 fill; Jacket Potato 2 Fil; Jacket potato 2 fill; Jacket Potato Deal; Large Roll; Large Roll Meal Deal; Mash/Veg/Beans; Meal Deal Jacket Pot; Meal Deal Pasta Dri; Meal Deal Sandwich; Meal Deal Soup & Rol; Oval Bun; Panini; Panini Deal; Panini Meal Deal; Paninin Deal; Paninis; Pasta & Dessert; Pasta Dish; Pasta Dish Meal Deal; Pasta Meal Deal; Salad Meal; Salad Meal & Dessert; Salad Meal Deal; Salad of the Day; Sandwich; Sandwich Meal Deal; Sandwich Stottie; Sauce portion; Sauce/Gravy/Curry; Small Soup Baguett; Small Stottie; Soup & Bread roll; Soup & Main Meal; Soup and Bread Roll; Soup of the Day; Soup with Roll; Special Roll; Standard Biscuits; Standard sandwich; Stottie; TrayBake; Triangular Sandwich; Veg/Salad; Vegetarian meal deal; Wrap; Wrap Meal Deal; Wraps	26 652	22 %
Insufficient detail to no code assigned	Dessert; Hot Pudding; Main & Dessert; Main 2 Cource Meal 1; Main 2 Course Meal 1; Main 2 Course Meal 2; Main 2 Course Meal 3; Main Dish; Main Meal & Desert; Main Meal & Dessert; Main Only; MD JP x1 Sm Drink; MD Main Sm Dr Pud; MD MC Sm Drink; MD ST Sand Sm Dr Bis; MEAL DEAL 1·90; MEAL DEAL 1·95; MEAL DEAL 2·00; Meal Deal Box; Misc; Open Food; Option 1; Option 1 meal deal; Option 2; Option 2 Meal Deal; Packed Lunch Deal; Packed Lunch Meal; Vegetairan; Water Meal Deal	20 954	18 %

NDNS, National Diet and Nutrition Survey.

* Intake24 food groups.

† NDNS nutrient databank code.

Eight percent (*n* 29) of item descriptions could not have a food group or NDB code assigned due to lack of information. These included items such as ‘Main Dish’ code assigned due to lack of information. These included items such as ‘Main Dish’, Main Meal and Dessert and Packed Lunch Deal. These accounted for 18 % (*n* 20 954) of total purchases across the 4-week menu cycle (Fig. 2; Table 1). The proportion of purchases that were assigned an NDB code ranged from 49 % in school 2 to 71 % in school 3 (Table 2). The proportion of purchases that were assigned a food group ranged from 66 % in school 2 to 90 % in school 5.

**Table 2.** The proportion of total purchases that were assigned a nutrient databank code and an Intake24 food group, by school

School	Total number of purchased items	Percentage of purchases assigned NDB nutrient code	Percentage of total purchases assigned Intake 24 food group
1	19 294	65	83
2	12 097	49	66
3	24 326	71	89
4	39 351	55	78
5	24 057	58	90

NDB, Nutrient Databank.

The percentage agreement between the two independent coders was 65 % for NDB nutrient codes and 90 % for food groups. Discrepancies in NDB codes occurred amongst fruit juice-based drinks. For example, ‘Fruit juice cup’ was assigned the NDB code ‘*Mixed fruit juice pasteurised’* by the first coder and *‘Fruit flavour drink, no juice, ready to drink’* by the second coder. Although there was very good agreement for food groups, discrepancies were evident for some drinks, for example, ‘Flavoured water’ was assigned the food group ‘*Water*’ by the first coder and ‘*Other carbonated drinks (not diet)*’ by the second coder.

## Discussion

The findings indicate that SFPD is a feasible approach for assessing dietary intakes in secondary school pupils, and the linkage to food groups and nutrient codes was possible for the majority of purchased items. Extensive data formatting was required to ensure consistency across the five datasets. There was variation in item descriptions due to different labels on individual school canteen till buttons; however, it was still possible to group the food and drink items into similar categories (see online supplementary material, Supplementary Material 1). The level of detail in the food and drink categories could be adapted depending on the information required and the purpose of the research question. In this study, hot meals were categorised into baguette, beef, burger, chicken, fish, ham/gammon/pork, sausage and vegetarian. These could be combined into an overall ‘hot meals’ category if the type of meal was not required.

### Potential for food group analysis

Overall, 82 % of total purchases were assigned an Intake24 food group. These food groups were developed and modified by researchers at HNERC, Newcastle University. They have been used in numerous food studies and are part of the Intake24 dietary recall tool^(19)^. Food group analysis allows intakes of similar foods to be quantified and dietary patterns explored. Food group analyses using SFPD would allow the combination of foods and drinks bought together to be explored, including differences in purchases by FSM and non-FSM pupils and across different year groups (years 7 to 11). These analyses would contribute to ongoing work exploring the factors influencing pupils’ food and drink choices and improving our understanding of the school environment, highlighting areas for potential intervention^(20–22)^.

In 2014, nutrient-based standards for UK school lunches were removed, leaving food-based standards only. These standards have been ‘designed to help children develop healthy eating habits, and ensure that they have the energy and nutrition they need to get the most from their whole school day’^(4)^. They include

- one or more portions of vegetables or salad as an accompaniment every day
- one or more portions of fruit every day
- a dessert containing at least 50 % fruit 2 or more times each week
- at least three different fruits and three different vegetables each week

Food group analysis of school food purchases at lunchtime could allow comparisons between pupils’ lunchtime purchases and government standards to be explored. This will highlight the effectiveness of the standards in encouraging children to eat a healthy diet at school and inform intervention development and potential policy changes.

### Potential for nutrient analysis

Seventy percent of food and drink item descriptions had enough detail to allow an NDB code to be allocated; this equated to 60 % of total purchases (Table 1). A crucial limitation is the lack of portion size information to allow nutrient content to be calculated and whether purchase equals consumption. There is scope for age-appropriate average portion sizes to be used, such as published average portion sizes^(23,24)^. Lambert *et al.* ^(25)^ explored the validity of purchase data as a method of monitoring school food intakes, and variations in portion sizes were assessed. Actual portions of eighty foods on the school menu were weighed and an average was calculated. The authors reported that the average actual portion sizes compared favourably with published portion data, used widely in dietary assessment, and therefore, this approach may offer a solution to the lack of portion sizes in SFPD. Lambert *et al.* ^(25)^ also compared observed food and drink purchases in the school canteen with items recorded in the purchase data and found a 96 % accuracy rating. However, this study was conducted at an all-boys school and a considerable time ago; therefore, the generalisability of the findings should be considered, along with, potential changes to food and drinks on offer in secondary schools. Overall, the findings from the present study along with previous literature show promise and support further exploration of the use of purchase data as a potential method of dietary assessment.

Variation in the proportion of purchased items linked to a food group and an NDB nutrient code was evident between schools (Table 2). For school 5, although 90 % of purchases were assigned a food group, only 58 % were assigned an NDB code. This was due to a large number of item descriptions such as ‘tray bake’, ‘sandwich’ and ‘pasta dish’ for which the food groups ‘cakes’, ‘cereal based savouries’ and ‘pasta’ were assigned, respectively, but no NDB code could be assigned due to a lack of information on the exact item. The lowest proportions were seen in school 2, where 49 % and 66 % of purchases were assigned an NDB code and food group, respectively. This was due to a high number of purchases with generic descriptions, i.e. ‘main 2 course meal’ and ‘packed lunch deal’, for which neither an NDB code nor a food group could be assigned. In a previous study, the till buttons used by canteen staff were amended to describe the purchased item in more detail; this may be a potential solution for the future development of the method^(16)^.

Inter-coder reliability was very good for food groups (90 %). Agreement was lower for NDB coding (65 %), which was expected due to the vagueness of some item descriptions and the array of possible nutrient codes in the NDB. The best practice would be to agree as a team on the most suitable NDB code and food group and be consistent throughout the data. Further information could be obtained from schools for specific items, for instance, the types of drinks, to ensure the most appropriate NDB code and food group is assigned.

### Strengths and limitations of using purchase data

The key advantage of using purchase data over dietary intake data is the quantity of data available. Our data captured a total of 3466 pupils, which was approximately 80 % of pupils attending the five schools. In addition, the data are objective and therefore less susceptible to the biases associated with self-reported intake data^(26)^. Data across the whole menu cycle can be obtained and information on the time of purchase allows purchasing patterns across the school day to be explored. FSM data enables comparisons between purchases made by pupils receiving FSM and those paying for lunches to be examined, potentially highlighting areas of inequalities among FSM pupils; a group that can often be underrepresented in research studies^(27)^.

Although SFPD can be useful in exploring food choices in secondary school pupils, only items purchased on school premises can be explored; information regarding packed lunches brought from home and items purchased from shops on the way to school are not included. There are limitations in its use as a dietary assessment method. Firstly, these data lack crucial information on portion sizes (as mentioned previously), cooking methods and additions, which are required for accurate nutrient coding. An absence of detailed information meant assumptions were made when assigning the most appropriate NDB nutrient code. For instance, the toast was coded as ‘BREAD, 50 % WHITE AND 50 % WHOLEMEAL FLOURS TOASTED’ as the specific type of bread was unknown. Cod in batter was coded as ‘COD IN BATTER FROZEN BAKED’, but for some schools, the fish may have been fried. Additions, such as butter or jam on toast, are unknown; however, butter is likely to have been offered and this could be added as an assumption. Eighteen percent of purchases did not have a food group or an NDB code assigned due to a lack of information in the item description (Table 1). However data on ‘meal deals’, ‘pack lunch deals’ is still of interest in understanding pupils’ purchasing decisions at school. Although meal types were assigned to the data (breakfast, break, lunch), no analysis was conducted on this. Pre-ordered lunches go through the tills in the morning period, and it was difficult to separate pre-ordered items and items purchased by pupils. This finding will be explored in future work. The types of and variation in purchases across the school day are important for intervention development, as evidence indicates pupils may purchase their lunch during breaktime, at which the availability of fruit and vegetables, may be limited^(28)^. As mentioned, the possibility of changing till buttons to provide more information in the data warrants consideration for future development.

Data could not be obtained for two of the schools due to technical issues at the time of download. This did not impact the project aims for this study; however, it is worth considering this as a potential challenge when designing a study using SFPD.

This explorative study has shown how purchase data can be used to provide an objective insight into pupils’ food and drink purchasing habits at school. The use of purchase data to assess food group intakes offers huge potential, both as a monitoring tool and as an evaluation method for school-based interventions aimed at improving food and drink choices^(16)^. The application is relevant beyond the UK, with cashless purchasing systems being used globally^(29,30)^. Future work comparing SFPD with self-reported food and drink intakes, such as dietary recalls, is needed to determine the level of agreement with an established dietary assessment method. However, there is scope for both methods to complement each other.

SFPD provides a novel, consistent approach to capturing school food intake data, from all secondary pupils, across the school day and across the socio-economic spectrum, enabling the development of large-scale interventions and policy evaluations, at both the local level and across regions. Furthermore, this work addresses key challenges of dietary assessment in adolescents and supports the need for novel dietary assessment methods^(10,11,31)^.

## Supporting information

Bradley and Spence supplementary material 1 Bradley and Spence supplementary material

Bradley and Spence supplementary material 2 Bradley and Spence supplementary material

## Supplementary material

For supplementary material accompanying this paper visit https://doi.org/10.1017/S1368980025000527
