# Supplementary material for: Exploring the feasibility of using school food purchase data as a method to assess dietary intakes in secondary school aged pupils
Source: Public Health Nutr. 2025 Apr 14;28(1):e82. doi: 10.1017/S1368980025000527 (PMC12100558; doi:10.1017/S1368980025000527)
Supplement: Bradley and Spence supplementary material 1 — Bradley and Spence supplementary material [file S1368980025000527sup001.docx]

Supplementary material 1

Table A: Item categories created and the item descriptions from the school food purchase data included within each category

| **Item category created by research team** | **Food and drink item descriptions in purchase data (derived from school canteen till buttons)** |
| --- | --- |
| Bacon/sausage bun | Bacon & Sausage Bun; Bacon Bun; Bacon Sandwich Large; Bacon Small; Breakfast Bun; Sausage bun |
| Bagel/crumpet/teacake/toast | Bagel; Bagel HALF; Cheese Scone; Croissants; Crossiants; Crumpet; Crumpets; Pancakes; Teacake; Toast; Toast & Butter; Toast Main Hall; Toasted Tea Cake |
| Biscuit | Assorted Biscuits; Biscuit; Choc bis; Homemade Biscuits; Standard Biscuits |
| Butter/marg | Butter/Flora Ptn; Extra Butter; Flora portion |
| Cake | Banana Cake; Banana Sponge; Carrot Cake; Chocolate Orang Cake; Ginger Cake; Iced Cake; Lemon Cake; Swiss Roll |
| cereal | Cereal; Porridge; Cereal Bars |
| Cheese | Cheese |
| Chips | Portion of Chips |
| Chocolate bar | Chocolate Bars |
| Crisps | Crisps; Pringles; propercrisps |
| Dessert | Dessert; Hot Pudding |
| Dessert cake | Apple Cake; Cake & Custard; Choc & Orange cake; Chocolate orange; chocolate sponge; Raspberry Coco Spong; Sponge Cake; Sticky Date Pud |
| Dessert crumble | Apple Crumbl; Apple Crumb; Apple Crumble; Cherry Crumble |
| Dessert dairy | Cheese Cakes; Eton Mess; Mousse; Rice Pudding; Strawberry Cheesecak |
| Dessert fruit flakes | Fruit Flakes |
| Dessert jelly | Jelly; Jelly Squeeze; Jellys |
| Dried fruit pot | Dried Fruit Pot |
| Fizzy drink | Coke; Dr Pepper; Fanta |
| Fizzy fruit juice | Radnor fizz; Radnor Fizz 300 ml; Suso Cans; Susu Can |
| Flapjack | Apple Flapjack; Berry Flapjack; Flapjack |
| Flavoured milk | Milkshake; Flavoured milk; Flavoured Milk 200ml; Milkshake |
| Flavoured water | Rad Water 330ml; Flavoured Water; Flavoured water 330m; Radnor Splash 500ml |
| Fruit juice | Carton Juice 220ml; Fresh fruit carton; Fruit Carton; Fruit Juice Carton; Fruit Juice Cup; Juice Burst; Juice Burst 330ml; Juiceburst; Juiceburst 330; Rad Frt Jc Car 125ml; Tropicana juice |
| Fruit juice drink | Cuplet; Oasis |
| Fruit pot | Fresh Fruit Pot; Fresh Fruit Salad; Fruit pot all; Fruit Salad; Melon fruit pot; Melon Pot; Pineapple fruit pot; Tub Grapes |
| Hot drink | Coffee; Hot Chocolate |
| Hot meal | Main Dish; Main Only; Pasta Arrabiata; Pasta Dish; Pasta Dish Meal Deal; Pasta with Sauce; Sweet n sour noodles; Sweet/Sour & Noodles |
| Hot meal baguette | Hot Meat Baguette |
| Hot meal beef | Bolognaise; Beef Stir Fry & Rice; Bolognaise & G/Bread; Chilli beef taco; Chilli Beef Tacos; chilli pasta; Chilli Taco; Chilli-Con-Carni; Cottage pie; Lasagne; Lasagne&Garlic Bread; Meatball Melt; Meatballs; Meatballs & Spag; Mince & dumpling; Mince & Dumplings; Mince and Veg; Mince Beef Casserole; Mince Pie; Roast Beef Dinner; Roast Beef&York/Pud; Spaghi Bolognese; Spicy Meat Balls |
| Hot meal chicken | Chicken Jalrezi; chicken wrap; BBQ Chicken; BBQ Chicken pizza; BBQ Chicken Wrap; Cajun Chick Quasadil; Cajun Chicken; Chick/Broc Pasta; Chicken Casserole; Chicken Curry; Chicken Dinner; Chicken fajita; Chicken Fajita Pocke; Chicken Fajitas; Chicken Italian; Chicken Jalfrazi; Chicken Korma; Chicken noodles; Chicken Pasta Bake; Chicken Tikka Masala; Chicken tomat pasta; Chicken tomato pasta; Chinese Chick Noodle; Harvest Chicken Cass; Italien Chicken; Jerk chicken; Roast Chicken Dinner; Roast Turkey; Roast Turkey Dinner; Sweet Chilli Enchil; Tikka Masala & Rice |
| Hot meal fish | Cod and Chips; Fish & Chips |
| Hot meal ham/gammon/pork | Gammon Pineapple; Gammon steak; Ham & Pin Pizza; Ham pizza; Roast Loin Pork; Roast Pork Dinner |
| Hot meal sausage | Cumberland sausage; Pork Sausage & Gravy; Sausage & onion; Sausage &Onion Gravy |
| Hot meal vegetarian | BBQ Veg Wrap; Beans on Toast; Cauliflower Cheese; Cheese & Tom Pizza; Cheese & Tom Quiche; Cheese &Onion Pie; Cheese Pizza; Cheese Quesadilla; Cheese tart; Cheese&Onion Quiche; Chinese noodles; Mushroom Korma; Pizza - Hidden Sauce; Quorn Korma; Quorn Lasagne; Spicy potato; Stuffed Peppers; Tomato Pasta; Veg Bolognaise; Veg Burger; Veg Chickpea Pocket; Veg Chinese Noodles; Veg Fajita; Veg Fajita Wrap; Veg Pea Pota Curry; Veg Spring Rolls; Vegetable Fajita Wra; vegetable wrap; Vegetairan; Vegetarian meal deal |
| Hot meal-burger | Burger; Grill Chicken Burger; Lamb Burger |
| Jacket potato | Jacket Potato 2 Fil; Bacon/Cheese Jacket; Baked Potato Deal; Filled Jacket 1 Fill; Half Jacket; Jacket & cold Fill; Jacket Meal Deal; Jacket Pot & Filling; Jacket Pot Meal Deal; Jacket Pot&2 Filling; Jacket Potato; Jacket potato 1 fill; Jacket potato 2 fill; Jacket Potato Deal; Jacket potato/butter; MD JP x1 Sm Drink; Meal Deal Jacket Pot |
| Jam portion | Jam portion |
| Meal deal | Main & Dessert; Main 2 Cource Meal 1; Main 2 Course Meal 1; Main 2 Course Meal 2; Main 2 Course Meal 3; Main Meal & Desert; Main Meal & Dessert; MD Main Sm Dr Pud; MD MC Sm Drink; MEAL DEAL 1.90; MEAL DEAL 1.95; MEAL DEAL 2.00; Meal Deal Box; Option 1; Option 1 meal deal; Option 2; Option 2 Meal Deal; Pasta & Dessert; Water Meal Deal |
| Milk | Milk; Milk carton |
| Misc | Misc; Open Food |
| Muffin | Lemon Muffin; Muffin |
| Noodle Box | Noodle Box |
| Packed lunch deal | Packed Lunch Deal; Packed Lunch Meal |
| Panini | Panini; Panini Deal; Panini Meal Deal; Paninin Deal; Paninis; Pepporoni Panini |
| Pasta pot | Meal Deal Pasta Dri; Pasta Meal Deal; Pasta pot; Pasta Pot Tomato; Pasta Pot Tuna; Plain Pasta; Plain Pasta & Cheese |
| Pizza snack | Cold Pizza Slice; Morning Pizza; Pepperoni; Pizza bread; Pizza Large; Pizza Muffin; Pizza Small; Pizzini |
| Popcorn | Proper Popcorn |
| Salad | Salad bar plate; Salad Bowl; Salad Meal; Salad Meal & Dessert; Salad Meal Deal; Salad of the Day; Salad Portion |
| Sandwich/baguette | Assorted Baguettes; Baguette; Baguette Meal Deal; Cheese Savoury; Cheese/Tuna; Chicken & Bacon; Chicken Tikka;  Classic Triangle S/W; Cold Baguettes; Ham; Large Roll; Large Roll Meal Deal; MD ST Sand Sm Dr Bis; Meal Deal Sandwich; Plain Chicken; S/F Chicken; Sandwich; Sandwich Meal Deal; Sandwich Stottie; Small Stottie; Standard sandwich; Sweet Chilli Chicken; Toastie; Triangular Sandwich |
| Sauces | BBQ Sauce; Extra Sauce; Ketchup; Mayo Sauce; Sauce portion; Sauce/Gravy/Curry; Sweet Chilli Sauces |
| Sausage roll | Sausage rolls |
| Soup/soup&roll | Homemade Soup; Meal Deal Soup & Rol; Small Soup Baguett; Soup & Bread roll; Soup & Main Meal; Soup and Bread Roll; Soup of the Day; Soup with Roll |
| Traybake | Chocolate Crunch; Cornflake Tart; Homebakes; TrayBake |
| Tuna | Tuna |
| Vegetable | Baked Beans; Extra veg portion; Mash/Veg/Beans; Sweetcorn; Veg/Salad |
| Water | Plain Water 330ml; Plain Water 500ml; Water; water 330ml |
| White bread | Bread bun; Crusty Roll; Garlic Bread; Oval Bun; Poppy Seed Large Rol; Special Roll; Stottie; Tandoori Flat Bread; Tandoori flatbread |
| Whole fruit | Fruit; Apple; Banana; Fresh Fruit; Fruit; Grapes; Melon; Orange |
| Wraps | Wrap; Wrap Meal Deal; Wraps |
| Yoghurt | Frozen Yoghurt; Yoghurt |
